# Examining and Contextualizing Approaches to Establish Policy Support Organizations – A Mixed Method Study

**DOI:** 10.34172/ijhpm.2021.86

**Published:** 2021-08-07

**Authors:** Sultana Al Sabahi, Michael G. Wilson, John N. Lavis, Fadi El-Jardali, Kaelan Moat

**Affiliations:** ^1^Centre of Studies and Research, Ministry of Health, Muscat, Oman.; ^2^Health Policy PhD Program, McMaster University, Hamilton, ON, Canada.; ^3^McMaster Health Forum, McMaster University, Hamilton, ON, Canada.; ^4^Department of Health Evidence and Impact, McMaster University, Hamilton, ON, Canada.; ^5^Centre for Health Economics and Policy Analysis, McMaster University, Hamilton, ON, Canada.; ^6^Department of Political Science, McMaster University, Hamilton, ON, Canada.; ^7^Africa Centre for Evidence, University of Johannesburg, Johannesburg, South Africa.; ^8^Knowledge to Policy Center, American University of Beirut, Beirut, Lebanon.

**Keywords:** Evidence-Informed Policy-Making, Knowledge Translation, Health Policy, Policy Support Organization

## Abstract

**Background:** There has been an increase in the number of policy support organizations (PSOs) that have been created to foster the systematic use of evidence in health system policymaking. Our aim was to identify approaches for establishing a PSO or similar entities by soliciting insights from those with practical experience with developing and operationalizing PSOs in real-world contexts.

**Methods:** We used a sequential mixed method approached. We first conducted a survey to identify the views and experiences of those who were directly involved in the establishment of PSOs that have been developed and implemented across a variety of political-, health- and research-system contexts. The survey findings were then used to develop a purposive sample of PSO leaders and refine an interview guide for interviews with them.

**Results:** We received 19 completed surveys from leaders of PSOs in countries across the WHO regions and that operate in different settings (eg, as independent organization or within a university or government department) and conducted interviews with 15 senior managers from nine PSOs. Our findings provide in-depth insights about approaches and strategies across four stages for establishing a PSO, which include: (*i*) building awareness for the PSO; (*ii*) developing the PSO; (*iii*) assessing the PSO to identify potential areas for enhancement; and (*iv*) supporting maturation to build sustainability in the long-term. Our findings provide rich insights about the process of establishing a PSO from leaders who have undertaken the process.

**Conclusion:** While all PSOs share the same objective in supporting evidence-informed policy-making (EIPM), there is no single approach that can be considered to be the most successful in establishing a PSO, and each country should identify the approach based on its context.

## Background

**Key Messages**

** Implications for policy makers**

- Availability of resources (eg, financial resources, or champion(s) who believe in the importance of evidence-informed policy-making [EIPM]) and identifying the gap in the system are good motivations to drive the establishment of a policy support organization (PSO).
- Conducting a situation analysis is an essential strategy in the process of establishing a PSO to raise awareness about the importance of EIPM, gain support from policy-makers, researchers, and stakeholders, identify the gap in the system, and understand who is doing what, how and why.
- Institutionalization of EIPM culture is essential for the sustainability of a PSO.

** Implications for the public**

Many organizations around the world solely focused on combining public opinions with other types of evidence (eg, research finding, data, and expert opinions) to support making the best policy that is efficient and accepted. These organizations aim to make the policy-making process more systematic and transparent to maintain the trust between the public and the government. These organizations aim to maximize benefits from other countries’ success and failure before introducing any program or policy into the home country. Taking this approach reduces the waste of limited resources, helps in selecting the most efficient programs’ and policies’ indicators, hence making the process of monitoring and evaluation more realistic and valuable in identifying the defect and reverse it with timely intervention.

Policy-making is a complex process where evidence is one factor among many that shape policy-makers’ decisions and actions.^1^ Policies informed by evidence are likely to be more effective and less expensive than policies formulated without it.^2^ Therefore, evidence-informed policies give policy-makers confidence in the decisions they make.^2^ Evidence can play an important role in clarifying a problem, framing viable options to address a problem, identifying implementation considerations and developing monitoring and evaluation plans that enable rapid-cycle improvements over time to implemented polices.^3-5^ Furthermore, evidence can also be used to understand the contextual factors that influence the selection of particular policy options.^3^

 Despite the importance and benefits of evidence-informed policy-making (EIPM), several barriers constrain the use of research evidence in health-system policy-making processes. Specifically, barriers can include limited access to high-quality and relevant research evidence, poor communication between policy-makers and researchers, combined with the complex nature of policy-making where evidence needs to be considered alongside institutional factors, pressure from interest groups and often competing political and societal values.^6,7^

 Over the last decade EIPM has gained global attention and a large body of literature from both academic and policy communities has emerged around how to address the challenges of supporting EIPM. Accordingly, there has been an increase in the number of policy support organizations (PSOs) that have been created to link research to policy and to foster the systematic use of research evidence in health systems policy-making.^8-10^ Efforts from the World Health Organization (WHO) to establish such organizations started in 2005 through the creation of the global Evidence-Informed Policy Network to promote the systematic and transparent use of health research evidence in policy-making.^11,12^

 We know from the literature that typically, a PSO is a national or state-level entity that may take several different forms. It might be a web-based entity, a network that forms around a particular issue or an event, or it might have conventional office premises.^13,14^ A PSO can stand-alone, or be part of a government or academic institution.^9,12,15,16^ Each of these forms has a set of advantages and disadvantages. Standing alone, a PSO might have the advantage of neutrality and independence to successfully broker among different stakeholders; yet being independent might affect the organization’s financial stability.^9,12,15,16^ On the other hand, being part of government, is advantageous in understanding the policy-making process and in strengthening the capacity of policy-makers to access, assess, adapt and apply research evidence. However, this proximity to policy-makers may compromise the organization neutrality which is essential in EIPM.^9,12,15,16^

 The literature further identify that a PSO may offer various services. These services include facilitating meetings among multiple stakeholders (eg, citizen panels and stakeholder dialogues), identifying and documenting local research (eg, administering clearinghouses), synthesizing and packaging research evidence (eg, rapid responses and policy briefs), aligning research topics with policy needs (eg, priority-setting exercises), and strengthening the capacity of researchers (eg, to understand the policy process) and policy-makers (eg, to access, assess, adapt and apply research evidence).^9,12-20^

 Although the literature provides insight about the features of PSOs’ (eg, location and services offered) and some country-specific examples of establishing PSOs,^21-24^ there has been less emphasis on deriving context-specific insights that can be used to generate themes that may be more broadly applicable across a range of settings. We recently developed a conceptual framework about the process of establishing PSOs based on a critical interpretive synthesis.^25^ However, there is a need to build on this work to integrate these theoretical findings with real-world experiences to provide additional insight into approaches for establishing PSOs and identifying contextual factors that influence the organizations’ establishment and its features. To achieve this goal, we conducted a sequential mixed methods study to complement the conceptual framework we developed earlier. Specifically, our aim was to solicit real-world insights from multiple policy-support organizations that exist in different settings and have different features.

## Methods

 To develop a broad as well as in-depth understanding about the phenomenon of establishing PSO, we used a sequential mixed method approached by combining elements of quantitative (survey) and qualitative (key informant interviews) research approaches. We first conducted a survey to identify the views and experiences of those who were directly involved in the establishment of PSOs that have been developed and implemented across a variety of political-, health- and research-system contexts. The survey findings were then used to refine a sample and interview guide for the qualitative interview study that we conducted in the second phase of the study. The interviews focused on: (*i*) identifying insights about the process of establishing a PSO; (*ii*) identifying the critical junctures in the life of PSOs; and (*iii*) identifying the approaches that PSOs have used to ensure organizational sustainability in the long term. Taken together, the quantitative and qualitative components yield a better mapping of the current features and contexts of different PSOs, in-depth understanding of their story of the establishment, and provided us with an opportunity to identifying different approaches for establishing PSOs that were not country-specific, which wouldn’t have been possible through each method in isolation.

###  Phase One: Survey 

 A questionnaire was developed and piloted with two PSOs which operate in different political, health, and research system contexts. The goal of the pilot study was to test the clarity of the questions and estimate the time needed to complete the questionnaire. The questionnaire covered five domains: (*i*) the political-, health- and research-system contexts in which the PSO operates; (*ii*) the focus/scope of the organization’s work; (*iii*) the PSO’s activities and products; (*iv*) the PSO’s attributes; and (*v*) the establishment phase of the PSO. Twenty-eight of the 37 questions asked were close ended.

####  Sampling and Recruitment

 Purposive and snowball sampling techniques were used to identify the study population. The PSOs were purposively sampled to represent different political-, health-, and research-system contexts and demographic variables. The principal investigator (PI) generated a list of 57 potentially relevant organizations identified through electronic searches of peer-reviewed articles and organizations’ websites. The list was then reviewed by the research team which applied inclusion criteria, which resulted in 23 organizations being included in the sample. The inclusion criteria specified that for any organization to be eligible to be invited for the survey, it had to perform at least one of the following functions (or a closely related function): (*i*) produce systematic reviews, or other types of syntheses of research evidence in response to requests from policy-makers; (*ii*) identify and contextualize research evidence in response to requests from policy-makers; and (*iii*) plan, commission, or carry out evaluations of health policies in response to requests from policy-makers. The organizations were excluded if they receive core funding from industry, they only produce or provide health or healthcare utilization data, or those that do not primarily focus on translating research evidence for supporting health system policy-making (eg, clinical practice guideline or public health practice). In addition, social media was used to recruit organizations that were interested and eligible. The PI created a video to invite eligible organizations to participate in the study. The research team tweeted the video with the link to the survey. Finally, participants were asked to name similar organizations that would be eligible for the study.

####  Data Collection and Analysis

 LimeSurvey was used to administer the survey. The survey link was sent by email with an invitation letter to the directors or senior managers of each eligible organization. Three reminders were sent to non-respondents a week, two weeks, and three weeks after the initial contact.

 Survey data were summarized using simple descriptive statistics. The open-ended questions were analysed using a qualitative descriptive approach. The analysis was oriented toward summarizing the informational contents of that data regarding PSO features. Initially, the written comments were grouped by the questions to offer a comprehensive summary of the findings. While staying close to the data and to the surface of words and events, the summarized findings under each question were then coded further, and further modified in the course of analysis to reflect emerging themes and the research objectives.

###  Phase Two: Interviews 

 Semi-structured interviews were conducted using an interview guide with prompts focused on the establishment, critical juncture(s), and sustainability of the participant’s organization. The survey findings were used to inform the interview guide and it was iteratively revised as needed to allow for exploration of emerging themes and to validate assumptions or statements made by other participants.

####  Sampling and Recruitment 

 We purposively sampled PSOs to ensure a mix of those with different features of interest (eg, knowledge production vs. translation, extent of engagement with policy-makers, stakeholders and researchers, etc), that were located in high income countries vs. low- and middle-income countries and that were new (established within the last five years) vs. old (established within more than five years ago). Based on these criteria, we selected 10 PSOs from among the survey respondents. Once the final list was generated, the PI conducted three meetings with two research members (JNL and KM) who were familiar with the history of these organizations to provide additional context to use as interview prompts during the interviews. A summary of the sampling and recruitment strategy of both phases, the survey, and the interview can be found in Supplementary file 1.

####  Data Collection and Analysis

 All participants were contacted via email to schedule a time for a telephone, Skype, or face to face interview with invitation letter and consent form. The aim was to conduct one interview for each PSO. However, in instances where the initial interviewee for a PSO was not able to speak to all of the areas of interest or they recommended an additional contact to provide further details, one additional interview per organization was scheduled.

 In an earlier study we developed a framework that outlines the process of establishing PSOs.The framework includes four main stages: awareness, development, assessment, and maturation. The awareness stage provides the foundation for establishing a PSO by identifying the motivation that would advance the idea of establishing a PSO toward the development stage where the focus is on the actual implementation of a PSO. The assessment stage consists of monitoring and evaluating either specific programs and services provided by the PSO or its overall performance. Finally, the maturation stage focuses is ensuring sustainability over the long-term (More details about the framework can be found at Al Sabahi et al).^25^

 Using this framework, the data were analyzed using the constant comparative approach.^26^ The open coding was started by classifying information into five main sections, which included the four stages in the framework and the contextual factors that facilitate or hinder the establishment. For each section, a number of codes were assigned to the interview for each respondent. These codes were then organized into categories, and the categories organized into larger themes. The themes and categories were refined iteratively. The findings from the survey open-ended questions were incorporated in this analysis for developing the themes where relevant. After the analysis was completed, we conducted member checking by sending a draft of the results to participants who were asked to review the findings and provide feedback.

## Results

###  Survey Findings

 A total of 19 surveys were completed, which included 14 from the 23 organizational leaders who were directly invited to participate and five additional surveys from the promotion of the survey on Twitter. The sample of participating organizations, although not perfectly balanced, are based on different geographical regions in the world (based on WHO regions) and from countries of different economic level (see Table S1, Supplementary file 2).

 The participating organizations work in different political, health and research system contexts (see Table S1, Supplementary file 2). Although most organizations provide service at more than one jurisdictional level, the main focus of all the organizations is at the national and sub-national levels. In addition, most of the organizations operate in health systems that are mainly centralized (7/17), mainly publicly funded (12/18), and predominantly publicly delivered (11/18). Although more than two thirds of the organizations reported having centralized funds for health-system research, only half of the funding organizations prioritize knowledge translation (KT) activities and require collaboration with researchers and policy-makers. Neither the health system nor the research system arrangements seem to influence the jurisdictional level at which the organizations can provide service.

####  Organizational Characteristics 

 PSOs have unique features (see Table S2, Supplementary file 2) which are a critical enabler for their activities. Most PSOs indicated they are located as an independent organization (7/15) or in a university (5/15) with fewer embedded within government (3/15). However, slightly less than one-third of the organizations classified themselves as a government department and/or as having linkages with government institutions, which could be the reason for reporting the government as the most common source of funding (11/15). Having an executive board is the most common governance approach (9/15) across all regions, and (12/15) of the organizations have linkage with different organizations (with the most frequent collaborations being with governments and academic institutions). Organizational budgets ranged from $10 000 to $1.25 billion, however we suspect that those who reported very high budgets misinterpreted the question and provided the budget of the larger organizations in which they are embedded. Most organizations indicated that the most common background training of staff is in health services research and in population and public health research (13/15). For accessing needed research, most PSOs have access to more than one database. About two thirds of the organizations have a strategic plan and only half of them monitor and evaluate their impact and update their work irregularly. Another misinterpretation of the question may have occurred in relation to organizational age which was reported as ranging from 3-71 years, with those PSOs in larger organizations likely having reported that age instead of when their unit was established. For the purpose of establishing these organizations, participants reported that lessons from other organizations were helpful (8/15), one-third conducted a situation analysis (5/15) and few of them used a readiness assessment tool (1/15).

###  Organizational Focus, Activities, Products, and Target Audience Engagement

 Across the different WHO regions, all organizations provide services in multiple domains and engage in a wide array of activities (see Table S3, Supplementary file 2), and (14/17) of the organizations reported engaging in the four main areas of policy development (ie, clarifying problems, framing options, identifying implementation considerations and supporting monitoring and evaluation). The most common activities to pursue this work are supporting learning about how to make evidence-informed decisions (14/16) and synthesizing research evidence (15/16). The data reflected that slightly more than two-thirds of the organizations formulate recommendations and among those that do, the most popular approach is through formal and informal consensus (6/10). These activities coincide with the different products produced (see Table S4, Supplementary file 2) with the most commonly produced products being evidence briefs (14/16), rapid syntheses or rapid reviews (12/16), databases of research evidence (10/16) and information about the health system in which the organization works (10/16). Using these activities and products, PSOs usually target different audience using different strategies (see Table S5, Supplementary file 2). In particular, health system policy-makers were more often engaged in PSO activities such as reviewing reports’ drafts (15/16) and being a member of the organizational governance or participants in the organization’s activities (14/16). Despite the international call for engagement of citizens in producing research and in developing policies and programs, this is the least frequently targeted audience by PSOs. This is consistent with our survey which found that only (7/16) convene deliberation with the citizens.

####  How the Survey Was Used to Inform the Second Phase of the Study 

 The survey results clearly indicate that PSOs vary in their contextual features, organizational attributes, focus, activities, products, and target audience. No special trend or pattern was observed to dominate organizations from a particular region, economic level, or setting. However, the results emphasized the importance of selecting organizations that have a rich story to contribute to answering the main research question about the organization establishment approach. Accordingly, after the complete analysis of the survey and carefully reading the participants answers to the open ended questions, a list of the participating organizations was generated with their main distinctive characteristics (the list also had comments on the depth of the participants responses) to guide the purposeful sampling of the participants to be interviewed. The study did not aim to make a comparison between organizations with different features, setting, or context. The aim was to get an in-depth insight from the full spectrum of PSOs about their establishment approach.

###  Qualitative Interview Findings 

 Eleven interviews were completed with 15 senior managers from nine organizations. Interview length varied from 45 to 90 minutes with an average of 60 minutes. Ten of them were conducted via Skype and one was face-to-face. We interviewed participants from organizations in five of the six WHO regions with three from the Americas, two from Africa, two from the Eastern Mediterranean, one from Europe, and one from the Western Pacific region, but none from South-East Asia.

 We organize our findings below according to the four stages of developing a PSO (awareness, development, assessment, and maturation) that we used to structure our interview guide.In each section below we outline the common approaches and strategies used and actions taken for each stage were identified. This is followed by an analysis of the contextual factors that participants identified as being important for establishing a PSO, which includes a SWOT (Strengths, Weaknesses, Opportunities, and Threats) analysis.

####  Awareness Stage – Fostering the Conditions Needed to Establish A PSO 

 Our interviews revealed three common approaches that respondents identified as helpful to raise awareness about the need for establishing a PSO. We provide an overview of each in Table 1 along with illustrative quotes from the interviews. The first approach related to establishing a supportive climate among policy-makers and researchers to advocate for the importance and the advantages of using evidence in policy-making. Participants reported that conducting workshops, distributing information about KT (eg, books, CDs, or pamphlets), and conducting one-on-one meetings with policy-makers, or large-group meetings with researchers and policy-makers to raise the awareness about the EIPM were good starting points to establish a supportive climate for EIPM. Participants highlighted that these initiatives focused mainly on clarifying the concept of EIPM in order to reach consensus about what has to be done to support the use of evidence in policy-making and to build buy-in from policy-makers.

**Table 1:** Common Approaches and Strategies for the Awareness Stage of Establishing a Policy Support Organization

**Common Approaches **	**Establishing Supportive Climate **	**Making Adequate Resources Available **	**Addressing the Gap in the System **
Strategies	Government, universities, civil society organizations and NGOs demonstrate interest in providing research that can be used in policy-making (eg, working together to identify the problems facing the health system). Researchers prepare a vision and mission to expand the contribution of research in policy-making. Governments demonstrate interest in supporting EIPM (eg, support building capacities in health system research and considered this support as an investment for the government to achieve its goal). International organizations create awareness about and demonstrate the need for EIPM through meetings, conferences and publications.	International organizations provide financial and/or technical support for starting EIPM initiatives. Governments and/or the hosting organization provide monetary and/or non-monetary (eg, building, access to the internet and electronic databases) support for establishing the PSO. The hosting organization demonstrates the availability of the appropriate expertise to lead the initiative. The hosting organization has a mandate to build collaboration within the organization and/or across other organizations to strengthen the health system that it seeks to support.	The following strategies were identified as being used by participants to provide justification to local, national and international organizations to support establishing a PSO: ♦ increase the awareness about the fragmentation between research and policy communities; ♦ demonstrate the potential benefits in creating a platform that would bring together policy-makers and researchers and allow them to incorporate the perspectives of civil society; ♦ demonstrate the added value from the proposed initiative compare to the other already existing; and ♦ highlight the gap that needs to be addressed.
Example/ illustrative quotation(s)	*“There was an interest from the government and universities on how to increase the research capacities that will help in providing research that the government can use” *[Participant 1].	*“The MOH provide money to stimulate research that can be better used by government through the involvement of all universities in the state” *[Participant 1].	*“*There* was a gap, there were people support public health and clinical decision but nothing about the health system, so there was a gap in terms of what is offered. This creates demand and needs for a group like us to fill this gap” *[Participant 2].

Abbreviations: NGOs, non-governmental organization; EIPM, evidence-informed policy-making; PSO, policy support organization; MoH, Ministry of Health.

 Second, participants focused on activities for making adequate resources (ie, human resources, financial resources, and infrastructure) available to help researchers and policy-makers determine the feasibility of establishing such an organization. Through participants sharing their stories with establishing a PSO we found that the establishment was derived either by the availability of the financial resources from local, national or international organizations, or by the availability of champion(s) who believed in the importance of EIPM and have the interest, the skills, and the experience needed for establishing such entity, or both. Participants highlighted that attracting resources and highlighting this could increase policy-makers’ willingness to accept the idea for establishing a PSO.

 Lastly, participants highlighted activities for addressing the gap between research and policy communities and supporting the use of research evidence in policy-making. Participants highlighted two types of gaps in the system. The first is the gap between the producers (researchers) and users (policy-makers) of the research evidence. Mainly due to the poor communication between the policy and research communities, many participants indicated that policy-makers were not aware of the existence of evidence that might be useful for formulating policies. At the same time, researchers produce evidence that was more of an academic interest instead of focused on policy-makers’ needs. The second gap was related to what was offered as support for informing policy-making using evidence. For example, several participants pointed out that there was enough work in using evidence for developing clinical practice guidelines, health technology assessment, and public health practice, but not for informing decisions about the health systems that get the programs, services and products to people who need them. Participants emphasized that highlighting these gaps and the benefits of addressing them were used as a justification to express the need for establishing a PSO and gain support from the national and international organizations.

####  Development Stage – Understanding the Situation or Proving the Concept and Defining the Organization’s Activities 

 Participants identified three different approaches as part of the development stage for a PSO, which we present in Table 2 along with illustrative quotes from participants. The first approach focuses on understanding the situation *or proving the concept *for establishing the PSO. Most participants reported that they conducted a situation analysis before establishing their PSO using different tools (eg, SWOT analysis, stakeholder power analysis, feasibility assessment, situation analysis using WHO guideline). A situation analysis was noted as having been conducted to identify: the target audience, key players who might have an interest in the organization’s work and can advocate for it, competitors, model organizations, individuals who have power and could influence the process of establishing a PSO (whether in terms of material resources or political power), capacity constraints, priorities for the PSO, and potential partners. Furthermore, participants also reported that situation analysis was conducted to understand policy-makers’ and researchers’ views and experiences with supporting the use of evidence in policy-making.

**Table 2:** Common Approaches and Strategies for the Development Stage of Establishing a PSO

**Common Approaches **	**Understanding the Situation or Proving the Concept**	**Setting the Perspectives Strategically **	**Implementing Start-up Activities **
**Strategies **	Demonstrate a proof of the effectiveness of EIPM by running some activities that immediately inform government priorities and involve both researchers and policy-makers. Understand who is doing what, how, and why by conducting a SWOT, feasibility analysis, situation analysis, future perspective analysis, policy analysis, or stakeholder power analysis. Interview and/or survey policy-makers to understand their needs and the barriers facing them in using research. Understand the administrative formalities and the requirements that are needed to be met for approval by the hosting organization.	Identify a model initiative and the pioneers in the area to learn from them and get their support and guidance during the process of establishing a PSO. Develop a steering committee or board of directors that has representatives with different expertise (eg, health, management, finance). Set organizational governance or mechanisms of working in a way that allows for engaging policy and research communities more effectively (eg, having the flexibility to engage/invite the appropriate partners based on the need of the different activities and projects). Develop a strategic plan with a clear vision, a mission, an operational plan, and strategic objectives to guide the next steps for developing the organization. Clearly define the organization branding. Set target(s) and indicators of success.	Raise awareness, about PSO activities and how it can support EIPM, across policy and research communities. Build the capacities of researchers to synthesize evidence in a user-friendly format and for building the capacity of policy-makers to access, appraise, synthesize and use evidence. For example, this can be achieved through workshops, face-to-face or online courses, and disseminating materials and guidelines on how to develop a policy brief and conducting a systematic review. Build the capacities of the organization’s core team through mentorship and Master’s and PhD programs. Build collaboration and networking with people and/or other organizations that have an interest and willingness to support efforts to advance the PSO’s work. Engage those with authority and interest in the PSO throughout all stages of PSO development. Apply for all available funding opportunities at the national and international level.
**Example/ illustrative quotation(s)**	*“We started by identifying a priority topic that will resonate with all policy-makers and created our first KT products ( ie, briefing note and policy dialogue). This was the proof of concept about what we can do particularly for the skeptics who thought we may have a hidden agenda” *[ Participant 3].	*“We constructed a temporary steering committee which worked on the business plan, budgeting, identifying risk factors, developing the governance model, identifying the value, the vision and the mission” *[Participant 2].	*“When we shared the vision and the mandate of the institution with the champions from [the] MoH, they bought the idea and supported it, they provided an avenue for interactions and provided an opportunity for engagement” *[Participant 4].

Abbreviations: EIPM, evidence-informed policy-making; PSO, policy support organization; MoH, Ministry of Health; KT, knowledge translation; SWOT, Strengths, Weaknesses, Opportunities, and Threats.

 Some organizations complemented the situation analysis with a proof of concept to give a concrete example for the target audience about what the organization can do and how they can benefit from its services. Most participants reported that they used the findings from the situation analysis for setting the organization’s perspectives and plans.

 Second, strategic development was used to guide the subsequent steps for establishing the PSO. Participants that conducted a situation analysis reported that the findings were useful for carefully framing the organization’s perspectives. In addition, participants also reported that they constructed a temporary steering committee that formulated the organization’s vision, mission, and objectives. For some organizations, the members of the steering committee were structured in a way that allowed it to draw on the pre-existing expertise at the hosting organization. For example, one of the participants from a PSO that is part of a university, constructed a temporary steering committee that involved expert from the business school to help with developing the business plan, budgeting, and identifying potential risk factors. Other organizations selected the members in a way that ensured representatives from the policy and research communities were engaged to create an avenue for their communication during the early stages of the organization’s establishment.

 Lastly, participants identified approaches for implementing start-up activities for their PSO. The most common activities identified at the early stage of developing a PSO were for building the capacity of researchers to synthesize evidence in a user-friendly format and for building the capacity of policy-makers to access, appraise, synthesize and use evidence. Workshops, meetings, face-to-face or online courses were the most common methods used for building the capacities of these groups. Participants also emphasized the importance of applying for all available funding opportunities at the national and international levels and therefore, building the capacity of PSO staff on how to apply for grants was reported as being highly useful for attracting more funds. In addition, training for conducting or leading the different activities of the PSO (eg, writing policy briefs or rapid syntheses, facilitating stakeholder dialogues, administering a database/clearinghouse for research evidence) was noted as also being crucial for implementing start-up activities. Participants indicated that building capacity typically took the form of mentorship by an expert (eg, the PSO leader and/or an in-house expert or from other organization or country), workshops, courses, and by providing scholarships for graduate students engaged in the PSO.

####  Assessment Stage – Identifying Opportunities for Enhancing the PSO

 Once a PSO is developed and starts to function on a regular basis, the next step needs to focus on assessing the quality of its work and performance to support continued enhancement of the PSO. As outlined in Table 3, we identified three common approaches from participants to guide the assessment stage. First, participants emphasized the need to create a plan to know what to assess when, how, and why. This was identified as being essential to track the organization’s performance (either by comparing the organization performance with its previous year’s achievements, or by comparing it with other similar organizations, or both) to identify areas and mechanisms for improvement. Participants consistently linked robust assessment to the organization’s reputation and trustworthiness in order to sustaining the demand for PSO services. Some participants noted that their organizations have a clear assessment plan as part of their strategic plan, and they set some key performance indicators for each goal they wanted to achieve each year. Indicators identify by participants to assess the organization’s performance included tracking the number of products (eg, policy briefs, systematic reviews and rapid syntheses completed, policy dialogues and citizen panels convened, capacity building workshops provided), number of grants received, number of collaborations built, and media coverage.

**Table 3:** Common Approaches and Strategies for the Assessment Stage of Establishing a PSO

**Common Approaches **	**Planning for Assessment **	**Implementing Assessment Activities**	**Identifying Needed Changes (Critical Juncture)**
Strategies	If the organization does not already have one, develop a strategic plan that guides the organization maturation and expansion and be clear what the organization wants to achieve. Set a regular monitoring and evaluation approach for the strategic plan and the annual plan. Use a transparent approach for the different activities of the organization. For example, publishing a handbook on how the organization conducts rapid evidence syntheses to highlight how its work is systematic and transparent. Develop a template for evaluating each activity. Set key performance indicators to assess the organization process and outcomes.	Conduct surveys and interviews or allow for regular feedback with those using or engaged in the programs and services provided by the PSO to assess the quality of the work. Engage external experts who are familiar with the local context to evaluate the PSO. Use a tracking system to determine the impact of the PSO on policy issues that it supported. Use an organizational retreat to reflect on the PSO’s work and identify changes needed.	Possible changes identified by participants included: ♦ changing the location of the organization or institutionalizing it (eg, within a government or another host institution); ♦ expanding the scope of the target audience to broaden the reach of the PSO (eg, from policy-makers at the Ministry of Health to parliamentarians); ♦ expanding the scope of the organization’s focus (eg, from health only to other sectors); ♦ adding new programs and services to the PSO’s set of activities; and ♦ publishing about the PSO’s model and the quality and importance of its work (eg, based on findings from monitoring and evaluation).
Example/ illustrative quotation(s)	*“We were clear about what we wanted to do, for what reason, what the end result would be, and how we would measure it. We are working on how to measure the impact” *[Participant 2].	*“There is an ongoing quality control mechanism by continually reviewing and revising the programs” *[Participant 5].	*“As we progressed, we saw that another category of policy-maker that are very vital to the policy-making process are parliamentarians. Now we include them in our engagement to guide them to make evidence informed legislations” *[Participant 4].

Abbreviation: PSO, policy support organization.

 After creating an assessment plan, the next approach involves implementing the assessment activities to identify what is working and what needs to be improved. Participants indicated that the assessment of the organization took place either annually (by the end of each financial year), or after specific activities (eg, end of project, after the delivery of a training workshop, policy dialogue, or a citizen panel), or by the end of the funding term in the cases where the organization started as a pilot project funded by international organization. Participants frequently reported that the assessment was conducted by PSO staff. One of the participants indicated that their PSO invited an external team (from a neighbor country that has a similar organization) to assess their organization to avoid bias and to be more objective. However, they found that experience to be challenging because it was hard to explain exactly how the system works because the impact on decision-making may not be apparent to someone without intimate knowledge about how local policy-making processes work. For aspects related to target audience feedback, surveys and interviews were used by most of the participants.

 Lastly, following phases of assessment, participants emphasized the need to use the results to identify changes that could be made to position the organization to enhance its impacts and to ensure sustainability in the long run. The most frequently reported change to a PSO by participants after an assessment was for moving towards institutionalizing the organization (eg, from being a pilot project to be institutionalized in a pre-existing organization), expanding the organization’s audience (eg, involving the media), and expanding the organization’s scope of work (eg, from health systems to health and social systems). These efforts were highlighted as critical junctures in the organization’s work that led to a significant shift in its success (success was subjectively defined based on each organizations’ leader’s view, experience, and objectives).

####  Maturation Stage – Building Sustainability in the Long-term 

 Participants from all organizations reported facing a challenge that threatened its sustainability. In discussing these challenges, we identified three common approaches which were used (or planned to be used) to position PSOs to be more sustainable in the long-term (see Table 4). The first approach is sustaining sufficient funding from the hosting organization or other national and international organizations. All participants highlighted the importance of ensuring financial stability for the PSO. Most participants obtained government support using different methods. Some identified having an agreement with their government to contribute to the PSO annual budget for a predetermined amount of work (eg, systematic review, policy briefs, policy dialogue). Others started offering courses related to KT through contracts with their government to train their staff who can then provide internal support to policy-makers, and/or by offering in person or online course for local and international participants to raise some funding through course fees. Still others sought funding through contracts with stakeholders to provide regular services (eg, rapid syntheses, stakeholder dialogue and citizen panels) or project-based contracts to offer specific services.

**Table 4:** Common Approaches and Strategies at the Maturation Stage of Establishing a PSO

**Common Approaches **	**Sustaining Sufficient Funding **	**Sustaining Appropriate Capacities **	**Approaching Institutionalization **
Strategies	Maintain trusting relationships with government partners. Use the organization’s programs and services as a source of self-funding (eg, by offering courses and workshops for a fee). Secure long-term funding through projects from external sources (ie, diversify funding to avoid relying on funding from a single hosting organization). Advocate for a fixed budget for KT components in all grant proposals to contribute to sustaining the use of PSO’s services (eg, through dedicated budgets for disseminating findings, preparing briefing notes and convening policy dialogues).	Provide incentives to attract and retain the human resources needed to maintain and advance the PSO. Obtain fixed staff positions from the hosting organization to avoid turnover and the loss of skilled staff. Maintain a high reputation of the PSO’s team and work (eg, credibility of the staff and transparency in work). Continue raising awareness and sensitizing policy-makers about the PSO’s work, particularly in times of political instability, to ensure continued demand.	Re-adjust the organization’s priorities to accommodate changes to the health system. Institutionalize the PSO within an existing organization to leverage existing infrastructure and resources. Institutionalize collaborations and networks to avoid continual change in the direction of the work. Incorporate the types of products produced by PSOs (ie, those that are not peer-reviewed manuscripts) as part of the annual performance review for faculty members.
Example/ illustrative quotation(s)	*“We thought that if the Ministry didn’t help us with funding then we probably wouldn’t be able to continue, so we invested in a lot of energy in ensuring that the relationship with the Ministry was solid”* [Participant 1].	*“We have a sustainability plan, which has a focus on how we attract and retain staff, and how we ensure that the skills are inside the organization” *[Participant 5].	*“Moving the unit to the health technology department gave it a better opportunity to grow because it became more stable, institutionalized, and had concrete resources from the public budget” *[Participant 6].

Abbreviations: PSO, policy support organization; KT, knowledge translation.

 Next, attracting and retaining staff with the right skills was noted being a challenge that had to be addressed in many PSOs. Participants consistently noted the need to have incentives (eg, competitive salary to avoid been attracted by competitor organizations or offering tenure positions for faculty) to retain staff. Participants from PSOs embedded in an academic institution expressed the importance of linking the PSO with programs provided through the university (eg, Master’s and PhD) as an opportunity to engage and retain skilled individuals, as well as a mechanism to address staff shortage by hiring students as part-time employees. This is dependent, however, based on whether there are staff at the PSO that are able to supervise graduate students.

 Lastly, institutionalizing the PSO by making its work embedded in the target organizations’ norms, cultures and processes was identified as essential to sustain demand from policy-makers. Although institutionalization was reported to play an important role in PSO sustainability, it was conceptualized differently by participants. Some participants approached institutionalization by attaching or embedding the PSO in a pre-existing institution. This approach was reported to be useful in ensuring access to the basic needs for the organization to function (eg, physical space, access to technology and staff salaries). The reputation of the hosting organization was also reported as adding value to the PSO. Other participants focused on institutionalizing the organization’s work by making it part of the regular work of the target audience to sustain the use of the organization’s services. For example, some organizations incorporated the types of products produced by PSOs (ie, those that are not peer-reviewed manuscripts, such as rapid syntheses, policy briefs and/or thematic analyses of stakeholder dialogues) as part of the annual performance review for faculty members. Participants also reported the importance of institutionalizing collaborations and networks by making them formal (eg, signed contract or memorandum of understanding) and less dependent on a particular individual to avoid the discontinuity that can result from staff turnover from any organization.

 None of these approaches were found to dominate over the other. However, being mindful of how to hire and retain staff and having the flexibility to quickly readjust the organization’s priorities to accommodate the continual changes in the health system were the most common strategies recommended by participants to approach sustainability. Our findings also highlight that PSO sustainability is an issue regardless of the organization’s age, which emphasizes the importance of taking maturation into consideration as early as possible given that it heavily depends on the actions that take place in the earlier stages (eg, awareness about EIPM and the organization’s work, the organizational arrangements, and the quality of the organization’s works).

####  Contextual Factors

 Our interviews revealed a group of contextual factors that facilitated or hindered the establishment of PSOs throughout the four stages. Some of the factors are internal at the individual or organization level, while others were external (eg, at the system level). Table 5 provides a full list of these factors in a SWOT analysis format to distinguish between the internal and the external facilitators and barriers.

**Table 5:** Contextual Factors That Influence the Establishment Process

	**Strengths**	**Weaknesses**
***Internal***	Awareness about the fragmentation in the system and the importance of EIPM to address this fragmentation between research and policy communities. Strong relationships between the hosting organization government, academia, and/or societal groups. Extensive experience of a hosting organization with research and running different projects. Mandate of the hosting organization to incorporate evidence and/or work in collaboration with other sectors and institutions. Availability of the appropriate skillful human resources to lead the PSO and its programs and services. Institutionalization of the PSO. Close working relationships with those making policies to facilitate communication. Availability of start-up funds from governments, international organizations, the hosting organization, or a combination of different sources. Strong reputation of the PSO leader in the research and or policy communities, which facilitates communication and the understanding the needs of both sides.	Lack of clarity in the organization objectives, scope, and or approaches to enhance the use of research in policy-making. Having different expectations about the organization work from the research and policy communities. Insufficient funding, particularly at the start-up phase. Language issue in non-English speaking countries as most of the available resources to be synthesized are in English, and few people are interested to invest in learning other languages to support policy-makers. Poor infrastructure, particularly internet connection and access to electronic databases. Lack of sufficient human resources with the appropriate skills to support EIPM. High staff turnover. Staff overloaded with administrative work. Lack of awareness about the existence of the organization and the type of services can be provided.
	***Facilitators***	***Barriers***
	**Opportunities**	**Challenges/ Threats**
***External***	Publication of major reports that highlight how the health system could be better informed by evidence. Government interest in EIPM and willingness to invest in the initiative (eg, providing money and interest in collaborating and using the services provided). Previous regulations and policies that recommend strengthening the health system through EIPM and collaboration between research and policy communities. Government awareness and experience with EIPM (ie, the readiness of the political environment for such initiative) and willingness to work with researchers. High demand of evidence from different policy agencies. Existence of other organizations that support the use of evidence but in other areas (eg, for technical decisions about which drugs and technologies to fund). Existence of strong health research system (ie, fund, skilled researchers, electronic databases, research about the local context, collaboration with other research entities). Support from those with authority from the policy and/or research community to advocate for the PSO. Availability of financial and technical support from national and or international organizations. Reputation of the hosting organization (eg, to give the PSO the trust and neutrality).	Political instability (eg, elections, frequent change in the governments, war). Negative experience of policy-makers with researchers (eg, in how evidence has been synthesized and presented to them) and/or lack of interest in collaborating. Division of policy authority among multiple departments, organizations, or political levels that do not typically collaborate. Lack of awareness of the importance of the benefits of EIPM among policy and research communities. Week health research system (ie, lack of fund, skilled researchers, electronic databases, research about the local context, collaboration with other research interties) particularly health system research. Limited dissemination and communication of research findings. The need for a shift from prioritizing opinion from experts and/or interests to systematically and transparently synthesized research evidence. Limited political commitment. Cumbersome administrative processes to receive approval from a hosting organization.

Abbreviations: EIPM, evidence-informed policy-making; PSO, policy support organization.

 We found that the strengths and opportunities of some organizations were the weaknesses and threats of the others. Therefore, the organizations were capitalizing on the facilitators to mitigate the barriers they were facing. For instance, among the organizations that had a strength in the form of the availability of start-up funds (from governments, international organizations, the hosting organization, or a combination of different sources), many also faced the barrier of not having clear objectives, scope, and approaches to enhance the use of research in policy-making. In contrast, PSOs without sufficient start-up funds, expressed that they had clear objectives and a strategic plan that were framed by skillful personnel who were ready to lead the PSO. Consequently, this helped in attracting funds from local and international organizations. Similarly, many PSOs operating in a context of limited awareness about the importance of EIPM among policy and research communities, indicated that they capitalized on the international climate and publication of major reports (eg, SUPPORT Tools for evidence-informed health Policy-making) that highlight how the health system could be better informed by evidence.

 Because a PSO’s work is based on collaboration between different organizations and communities, it is important to take into consideration the facilitators and barriers that emerge at the individual, organization, and system level. A PSO should try to mobilize the weaknesses and threats to strengths and opportunities by considering the different strategies highlighted in the four stages. Although each factor listed in Table 5 has been reported by more than two organizations, there was no single facilitator nor barrier reported by all organizations simultaneously.

## Discussion

###  Principal Findings 

 We found that PSOs function at different political, health, and research system contexts and at more than one jurisdictional level. However, regardless of the contexts in which they operate, we found that the PSOs we surveyed provide services in multiple domains and engage in a wide array of activities.

 In our interviews with leaders of PSOs we derived in-depth insights approaches and strategies used to establish a PSO, as well as potential barriers and facilitators that can affect the process across the four stages of developing a PSO. Common approaches to establishing a PSO related to:

- building awareness for the need for a PSO (eg, establishing a supportive climate for EIPM, making adequate resources available, and demonstrating the conceivable role a PSO can play in addressing the gap between research and policy communities);
- developing the PSO (eg, understanding the situation where the organization would be established, setting the perspectives strategically to guide the subsequent steps for establishing the organization, and conducting the start-up activities to be able to function in a regular way);
- assessing the PSO to identify potential areas for enhancement (eg, planning for assessment, running the assessment activities to know what has been done appropriately and what needs to be improved, and considering the appropriate changes need to ensure sustainability in the long-term); and
- supporting maturation to build sustainability in the long-term (eg, ensuring sufficient funding; sustaining appropriate capacities by finding the right personnel, retaining them, and enhancing their skills; and approaching the institutionalization of the organization by making its work embedded in the target organizations’ norms, cultures, and processes).

We found that key barriers to this process included lack of funding, lack of human resources, and division of policy authorities. Key facilitators included awareness and government interest in EIPM, availability of support from national and international organizations, as well as at the individual level, organization level and system level. It is important to acknowledge that there were difference in the approaches and strategies used across contexts, and those working on establishing PSOs should be mindful about how these factors might differ in their context.

 By incorporating insight form PSOs’ leaders from around the world, our findings offer unique contributions to the literature. We provided lessons for establishing PSOs that are not country or region specific.^13,27-32^ Our study was inspired by the existing literature that focuses on identifying approaches to support EIPM (eg, setting the priorities, building capacities, and providing rapid response services) and the factors that might influence these approaches.^13,27-32^ Our study builds on this literature by providing insights about the process for establishing the organizations that support EIPM. While the literature highlighted the role of the government structure in the use of evidence in the policy-making process,^33,34^ our study was unable to provide evidence that supports any particular conclusion regarding the role of government structures in establishing PSOs. This might have three possible explanations which will need further investigation. First, there is a possibility that participants were not aware of the exact role of the government structure in establishing PSOs, either because they do not consider it as an important factor, or because it was not obvious enough to be considered as an important factor. The second potential explanation is that the diffusion of ideas about the importance of EIPM and establishing a KTP was mainly pushed by international organizations (many of the participants mentioned the support from the international organization). As a result, the some may have viewed government structures as not relevant given the more important influence from international organizations that influenced the actions of those structures. The last potential explanation is that the government structure was not an important factor for establishing PSO.

 The findings of this study help to enrich the framework we developed in an earlier study that illustrated the process of establishing a PSO by providing more detailed approaches and strategies that can be used in each stage.

###  Strengths and Limitations

 A key strength of our study was the use of a sequential mixed method approach. Conducting the survey first allowed us to document general PSO characteristics and then use those characteristics to purposively select a sample of PSOs that would provide rich insights from different regions of the world, from PSOs operating in different settings and from those which have demonstrated sustainability over the long-term and that have been recently established. In addition, our approach allowed us to confirm, triangulate and derive additional insights from the survey findings, which provides for a much richer set of data. A potential limitation of our study is the small sample size, which did not allow us to reliably identify patterns in establishment approaches across different contexts, regions, or organizational settings. However, this limitation is unavoidable given the limited number of PSOs that exist.

###  Implications for Policy and Research 

 The main implication of our findings is that they provide rich insights about the process of establishing a PSO from leaders who have undertaken the process. As a result, the stages of establishment presented in this paper and the insights about them from leaders of PSOs can provide important guidance for those who are considering establishing a PSO or who are already in the process of doing so. For example, our findings revealed the importance of conducting a situation analysis, and those who are interested in establishing a PSO should consider undertaking a similar process to enable them to develop the most appropriate strategy according to the context in which they work. In addition, our finding can be informative for leaders of PSOs to expand or refine their scope of work, such as by selecting a new program or service to provide and refining monitoring and evaluation plans to include assessments of the impact of their work.

 Future research could include conducting: (*i*) a larger-scale study to identify patterns in PSO establishment approaches, (*ii*) co-design studies that document and provide in-depth insights about the establishment of one or more PSOs; (*iii*) evaluations of the impact of PSOs on supporting EIPM; (*iv*) apply broader categories and themes to a specific context to determine whether they can be useful in understanding the process, and whether these themes can facilitate cross-context comparisons of setting up PSOs.

## Ethical issues

 This project was approved by Hamilton Integrated Research Ethics Board (HiREB) under the project ID 5377.

## Competing interests

 Authors declare that they have no competing interests.

## Authors’ contributions

 SAS was responsible for the conception and design, collection of data, analysis and interpretation of data, drafting and reviewing the manuscript. MGW contributed to the conception and design, analysis and interpretation, drafting and reviewing the manuscript. MGW was also responsible for supervising this work. JNL, FEJ and KM provided critical revisions of the manuscript for important intellectual content.

## Disclaimer

 The views expressed in this paper represent the opinions of the authors and not an official position of the institutions of their affiliation.

## Supplementary files

Supplementary file 1. Sampling and Recruitment for the Survey and Interview.

Supplementary file 2 contains Tables S1-S5.
