# Supplementary material for: Examining and Contextualizing Approaches to Establish Policy Support Organizations – A Mixed Method Study
Source: Int J Health Policy Manag. 2021 Aug 7;11(9):1788–800. doi: 10.34172/ijhpm.2021.86 (PMC9808249; doi:10.34172/ijhpm.2021.86)

**Article title:** Examining and Contextualizing Approaches to Establish Policy Support Organizations – A Mixed Method Study

**Journal name:** International Journal of Health Policy and Management (IJHPM)

**Authors' information:** Sultana Al Sabahi[1]*, Michael G. Wilson[2,3,4], John N. Lavis[2,3,4,5,6,7], Fadi El-Jardali[8], Kaelan Moat[3]

[1]Centre of Studies and Research, Ministry of Health, Muscat, Oman.

[2]Health Policy PhD Program, McMaster University, Hamilton, ON, Canada.

[3]McMaster Health Forum, McMaster University, Hamilton, ON, Canada.

[4]Department of Health Evidence and Impact, McMaster University, Hamilton, ON, Canada.

[5]Centre for Health Economics and Policy Analysis, McMaster University, Hamilton, ON, Canada.

[6]Department of Political Science, McMaster University, Hamilton, ON, Canada.

[7]Africa Centre for Evidence, University of Johannesburg, Johannesburg, South Africa.

[8]Knowledge to Policy Center, American University of Beirut, Beirut, Lebanon.

(*Corresponding authors: Al-sabahiS@hotmail.com)

**Supplementary file 1.** Sampling and Recruitment for the Survey and Interview

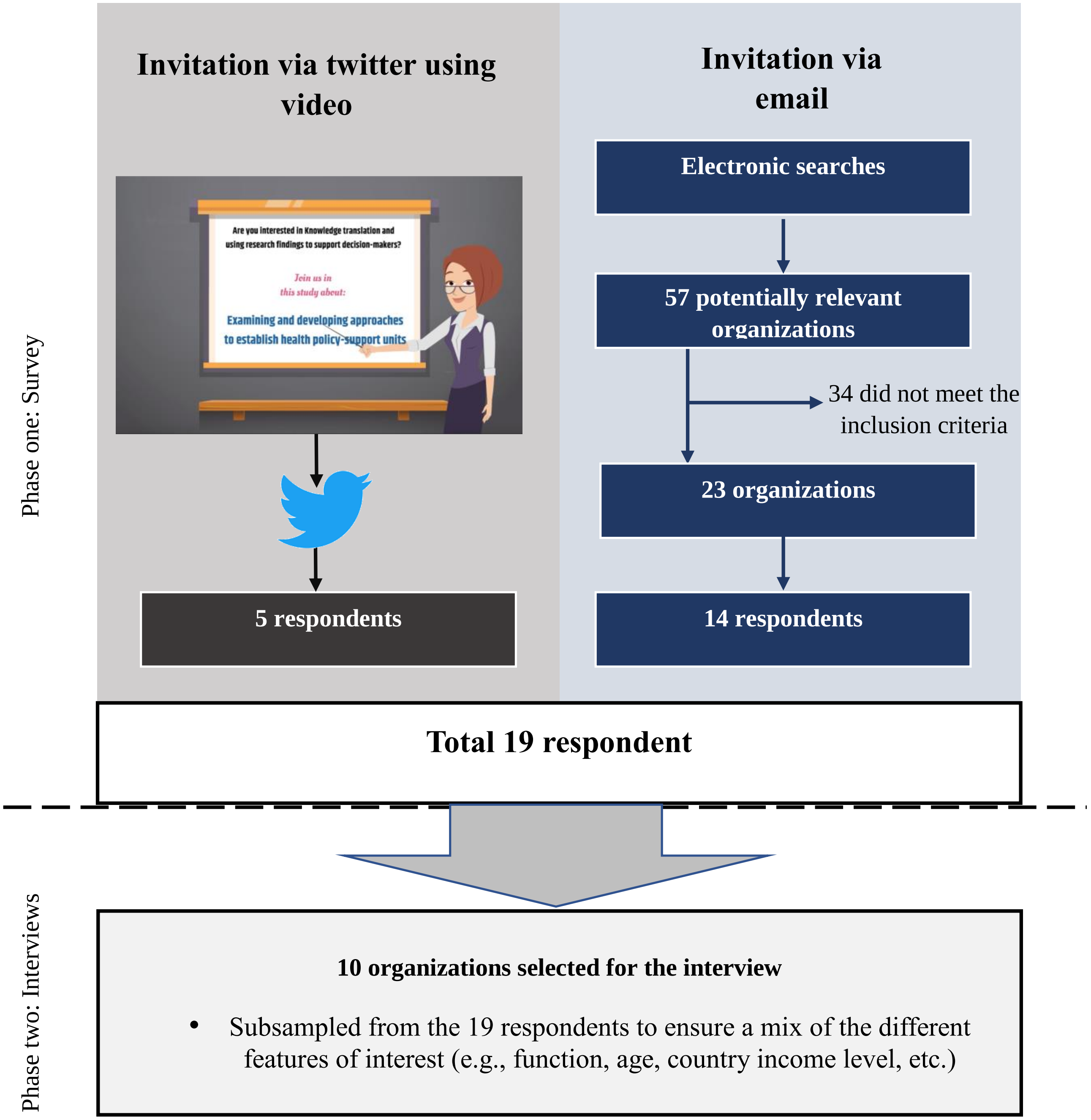

Supplement: Supplementary file 1 — Sampling and Recruitment for the Survey and Interview. [file ijhpm-11-1788-s001.pdf]
