# Supplementary material for: Examining and Contextualizing Approaches to Establish Policy Support Organizations – A Mixed Method Study
Source: Int J Health Policy Manag. 2021 Aug 7;11(9):1788–800. doi: 10.34172/ijhpm.2021.86 (PMC9808249; doi:10.34172/ijhpm.2021.86)
Supplement: Supplementary file 2 — contains Tables S1-S5. [file ijhpm-11-1788-s002.pdf]

Article title: Examining and Contextualizing Approaches to Establish Policy Support Organizations – A Mixed Method Study

Journal name: International Journal of Health Policy and Management (IJHPM)

Authors' information: Sultana Al Sabahi[1]*, Michael G. Wilson[2,3,4], John N. Lavis[2,3,4,5,6,7], Fadi El-Jardali[8], Kaelan Moat[3]

[1]Centre of Studies and Research, Ministry of Health, Muscat, Oman.

[2]Health Policy PhD Program, McMaster University, Hamilton, ON, Canada.

[3]McMaster Health Forum, McMaster University, Hamilton, ON, Canada.

[4]Department of Health Evidence and Impact, McMaster University, Hamilton, ON, Canada.

[5]Centre for Health Economics and Policy Analysis, McMaster University, Hamilton, ON, Canada.

[6]Department of Political Science, McMaster University, Hamilton, ON, Canada.

[7]Africa Centre for Evidence, University of Johannesburg, Johannesburg, South Africa.

[8]Knowledge to Policy Center, American University of Beirut, Beirut, Lebanon.

(*Corresponding authors: Al-sabahiS@hotmail.com)

Supplementary file 2.

Table S1. Contextual Feature of the of the Participated Organizations

	Total	WHO region				
		**Africa**	**America**	**Eastern Mediterranean**	**Europe**	**Western Pacific**
**Income level according to World Bank classification (n=18)**						
Low-income	3 (17%)	3 (17%)	0	0	0	0
Lower-middle-income	5 (28%)	3 (17%)	0	0	2 (11%)	0
Upper-middle-income	7 (39%)	0	3 (17%)	2 (11%)	2 (11%)	0
High-income	3 (17%)	0	2 (11%)	0	0	1 (6%)
***Political jurisdiction in which the organizations principally provide supports within (n=18)**						
National/ federal	17 (94%)	7 (39%)	4 (22%)	2 (11%)	3 (17%)	1 (6%)
Provincial/ sub-national	9 (50%)	3 (17%)	3 (17%)	1 (6%)	1 (6%)	1 (6%)
Local	5 (28%)	2 (11%)	2 (11%)	0	0	1 (6%)
Other (global)	1 (6%)	0	1 (6%)	0	0	0
**Governance arrangement of the health system in which the organizations operate (n = 17)**						
Mainly centralized	7 (41%)	0	1 (6%)	1 (6%)	4 (24%)	1 (6%)
Mainly decentralized	6 (35%)	2 (12%)	3 (18%)	1 (6%)	0	0
Equally centralized and decentralized	4 (24%)	3 (18%)	1 (6%)	0	0	0
**Financial arrangement of the health system in which the organizations operate (n = 18)**						
Mainly publicly funded	12 (67%)	5 (28%)	3 (17%)	0	3 (17%)	1 (6%)
Mainly privately funded	1 (6%)	0	0	1 (6%)	0	0
Mix of public and private	5 (28%)	1 (6%)	2 (11%)	1 (6%)	1 (6%)	0
**Delivery arrangement of the health system in which the organizations operate (n = 18)**						
Predominately public delivery	11 (61%)	4 (22%)	3 (17%)	0	3 (17%)	1 (6%)
Predominately private for-profit	1 (6%)	0	0	0	1 (6%)	0

Predominately private not-for-profit	1 (6%)	0	1 (6%)	0	0	0
Mix of private for-profit and not-for-profit	4 (22%)	2 (11%)	1 (6%)	1 (6%)	0	0
Equally delivered by public and private	1 (6%)	0	0	1 (6%)	0	0
**◊Research system in which the organizations operate (n = 18)**						
Availability of centralized fund for HSR	14 (78%)	4 (22%)	5 (28%)	1 (6%)	3 (17%)	1 (6%)
Provision of funds for prioritized KT activities from funding agencies	10 (56%)	3 (17%)	3 (17%)	0	3 (17%)	1 (6%)
Funding agency requirement for collaboration between researchers & policy-makers	10 (56%)	3 (17%)	3 (17%)	0	3 (17%)	1 (6%)
Funding agency requirement for collaboration between researchers & citizens	6 (33%)	1 (6%)	2 (11%)	0	3 (17%)	0

** More than one answer was possible for this question*

◊ Each question of this section has been asked separately

Note: the total percentage may not be exact because of the rounding

Table S2. Organizational Attributes

	Total	♦WHO region				
		**Africa**	**America**	**Eastern Mediterranean**	**Europe**	**Western Pacific**
**Location (n= 15)**						
Embedded within universities	5 (33%)	2 (13%)	1 (7%)	2 (13%)	0	0
Embedded within ministry of health	3 (20%)	0	3 (20%)	0	0	0
Independent	7 (47%)	1 (7%)	1 (7%)	0	4 (27%)	1 (7%)

**Organization type (n =14)**						
Government department/ unit	4 (29%)	1 (7%)	2 (14%)	0	1 (7%)	0
Research center in academic institution	2 (14%)	0	0	2 (14%)	0	0
Independent research center	3 (21%)	1 (7%)	2 (14%)	0	0	0
Evidence-Informed Policy Network	3 (21%)	1 (7%)	0	0	2 (14%)	0
Professional association	1 (7%)	0	1 (7%)	0	0	0
Independent non for-profit organization	1 (7%)	0	0	0	0	1 (7%)
**Governance approach (n =15)**						
Through executive board	9 (60%)	3 (20%)	2 (13%)	1 (7%)	2 (13%)	1 (7%)
Advisory committee	4 (27%)	0	1 (7%)	1 (7%)	2 (13%)	0
Other (in house senior officials)	2 (13%)	0	2 (13%)	0	0	0
**Budget in US dollar (n=12)**						
Mean	$ 105,880,000	$ 157,500	$ 312,820,000	$ 20,000	$ 1,975,000	13,000,000
Median	$ 250,000	$ 157,500	$ 62,5000	$ 20,000	$ 1,200,000	13,000,000
Range	$ 10,000 to 1,250,000,000	$ 65,000 to 250,000	$ 30,000 to 1,250,000,000	$ 10,000 to 30,000	$ 225,000 to 4,500,000	-
***Source(s) of funding (n =15)**						
Government	11 (73%)	2 (13%)	4 (27%)	1 (7%)	3 (20%)	1 (7%)
Grants from research-funding agencies	10 (67%)	2 (13%)	4 (27%)	2 (13%)	1 (7%)	1 (7%)
Philanthropic donations	1 (7%)	0	1 (7%)	0	0	0
International agencies	5 (33%)	1 (7%)	2 (13%)	0	2 (13%)	0
Biomedical or other for-profit companies	1 (7%)	0	0	0	1 (7%)	0

Other sources		4 (27%)	0	1 (7%)	1 (7%)	1 (7%)	1 (7%)
**Human resources (n =15)**							
Total number of staff	Mean	63	9	36	11	153	100
	Median	15	8	12	11	115	100
	Range	4 to 360	5 to 15	4 to 93	10 to 12	24 to 360	-
Full time employees	Mean	51	4	19	9	141	80
	Median	12	2	12	9	105	80
	Range	0 to 337	0 to 10	1 to 55	-	18 to 337	-
Part-time employees	Mean	14	5	23	2	13	20
	Median	6	5	9	2	13	20
	Range	0 to 66	3 to 8	0 to 66	1 to 3	0 to 25	-
***Background of full-time employees (n =15)**							
Policy analysis		12 (80%)	1 (7%)	4 (27%)	2 (13%)	4 (27%)	1 (7%)
Health economics		7 (47%)	1 (7%)	4 (27%)	0	2 (13%)	0
Health services research		13 (87%)	2 (13%)	4 (27%)	2 (13%)	4 (27%)	1 (7%)
Clinical epidemiology		10 (67%)	1 (7%)	5 (33%)	1 (7%)	2 (13%)	1 (7%)
Informatics / library science		5 (33%)	1 (7%)	2 (13%)	1 (7%)	1 (7%)	0
Biostatistics		9 (60%)	1 (7%)	4 (27%)	2 (13%)	1 (7%)	1 (7%)
Population and public health research		13 (87%)	2 (13%)	5 (33%)	2 (13%)	3 (20%)	1 (7%)
Other types of social science		7 (47%)	0	3 (20%)	1 (7%)	2 (13%)	1 (7%)
***Information system access (n =15)**							
Electronic databases		12 (80%)	1 (7%)	5 (33%)	2 (13%)	3 (20%)	1 (7%)
Database that index local studies or reports		9 (60%)	2 (13%)	4 (27%)	2 (13%)	1 (7%)	0
Local data sets		13 (87%)	2 (13%)	5 (33%)	1 (7%)	4 (27%)	1 (7%)

Other	1 (7%)	0	0	0	1 (7%)	0
***Organizational linkages (n =15)**						
**Type of organizational linkages**						
Government institution	11 (73%)	3 (20%)	3 (20%)	1 (7%)	3 (20%)	1 (7%)
Academic institution	12 (80%)	3 (20%)	5 (33%)	0	3 (20%)	1 (7%)
Other local/ provincial organization	8 (53%)	2 (13%)	3 (20%)	0	3 (20%)	0
Other international organization	11 (73%)	3 (20%)	3 (20%)	1 (7%)	3 (20%)	1 (7%)
Other (NGOs)	1 (7%)	0	0	0	1 (7%)	0
**Organizations that mentored others**	11 (73%)	2 (13%)	4 (27%)	1 (7%)	4 (27%)	0
**Organizations that have ‘sister’ centres**	3 (20%)	1 (7%)	1 (7%)	1 (7%)	0	0
**Organizations that have a strategic plan**	10 (67%)	0	3 (20%)	2 (13%)	4 (27%)	1 (7%)
**Organizations that monitoring and devaluation the organization performance/ impact**	8 (53%)	0	2 (13%)	2 (13%)	3 (20%)	1 (7%)
**Frequency update of products produced (n = 15)**						
Update regularly	2 (13%)	1 (7%)	0	0	1 (7%)	0
Update irregularly	8 (53%)	2 (13%)	3 (20%)	1 (7%)	1 (7%)	1 (7%)
Do not update	5 (33%)	0	2 (13%)	1 (7%)	2 (13%)	0
**◊ Establishment (n = 15)**						
Organizational age — Mean	23	20	24	8	31	17
Organizational age — Median	17	14	10	8	25	17
Organizational age — Range	3 to 71	4 to 43	3 to 60	4 to 11	2 to 71	-
Organizations that found lessons from other countries helpful	8 (53%)	2 (13%)	3 (20%)	1 (7%)	2 (13%)	0
Organizations that conduct situation analysis	5 (33%)	0	1 (7%)	2 (13%)	1 (7%)	1 (7%)

Organizations that used readiness assessment tool	1 (7%)	0	0	1 (7%)	0	0

♦ *The Western Pacific region dropped because the survey not complete*

* *More than one answer was possible for this question*

◊ *Each question of this section has been asked separately*

Table S3. Organizational Focus, and Activities

	Total	WHO region				
		Africa	America	Eastern Mediterranean	Europe	Western Pacific
***The domains in which the organizations provide service (n = 17)**						
Clarifying problems	16 (94%)	4 (24%)	5 (29%)	2 (12%)	4 (24%)	1 (6%)
Framing options	15 (88%)	4 (24%)	5 (29%)	2 (12%)	3 (18%)	1 (6%)
Identifying implementation considerations	14 (82%)	3 (18%)	5 (29%)	2 (12%)	3 (18%)	1 (6%)
Supporting monitoring and evaluation	14 (82%)	4 (24%)	3 (1918	2 (12%)	4 (24%)	1 (6%)
***Organizational activities (n = 16)**						
Provide online courses	6 (38%)	0	2 (13%)	1 (6%)	2 (13%)	1 (6%)
Provide face-to-face courses/ workshops	15 (94%)	4 (25%)	5 (31%)	2 (13%)	3 (19%)	1 (6%)
Provide searchable repositories/databases	8 (50%)	1 (6%)	4 (25%)	1 (6%)	2 (13%)	0
Conduct rapid synthesis/rapid reviews of evidence	12 (75%)	2 (13%)	5 (31%)	2 (13%)	2 (13%)	1 (6%)
Conduct systematic reviews of primary research	8 (50%)	3 (19%)	3 (19%)	2 (13%)	0	0
Conduct overviews of systematic reviews	8 (50%)	2 (13%)	3 (19%)	1 (6%)	2 (13%)	0
Create evidence/policy briefs	14 (88%)	3 (19%)	5 (31%)	2 (13%)	3 (19%)	1 (6%)
Convening deliberations with citizens/patients	7 (44%)	1 (6%)	3 (19%)	1 (6%)	2 (13%)	0

Convening deliberations among system leaders	13 (81%)	3 (19%)	3 (19%)	2 (13%)	4 (25%)	1 (6%)
Convening communities of practice	6 (38%)	1 (6%)	2 (13%)	0	2 (13%)	1 (6%)
Supportive audits of policies and practices	9 (56%)	1 (6%)	3 (19%)	2 (13%)	2 (13%)	1 (6%)
External reviews of reports or policies	11 (69%)	3 (19%)	3 (19%)	1 (6%)	4 (25%)	0
Adapting resources to the needs of the organization	10 (63%)	2 (13%)	3 (19%)	1 (6%)	3 (19%)	1 (6%)
Evaluate approaches to support EIDM	10 (63%)	3 (19%)	3 (19%)	1 (6%)	2 (13%)	1 (6%)
Other	3 (19%)	1 (6%)	1 (6%)	1 (6%)	0	0
**Organizations that formulate recommendations (n=14)**	10 (71%)	2 (14%)	2 (14%)	2 (14%)	3 (21%)	1 (7%)
***Methods of formulating recommendation (n = 10)**						
Subjective review	5 (50%)	1 (10%)	0	1 (10%)	2 (20%)	1 (10%)
Informal consensus	6 (60%)	1 (10%)	1 (10%)	2 (20%)	2 (20%)	0
Formal consensus	6 (60%)	1 (10%)	0	1 (10%)	3 (30%)	1 (10%)
Graded according to the quality	3 (30%)	0	1 (10%)	0	1 (10%)	1 (10%)
Other	1 (10%)	0	0	1 (10%)	0	0

* *More than one answer was possible for this question*

◊ *Each question of this section has been asked separately*

Table S4. Organizational Products

♣**Type of product**	**Number /16**	**Years since start producing this product**	**Number produced in the last 12 months**

		Median	Range	Median	Range
Rapid syntheses or rapid review	12 (75%)	4.5	1-12	5.5	0-46
Evidence or policy briefs	14 (88%)	5	1-10	2.5	1-10
Citizen friendly syntheses or summaries of research	8 (50%)	4.5	1-10	2.5	0-15
Analysis or summaries of deliberations	8 (50%)	7	5-10	2	0-20
Database of research evidence	10 (62%)	5.5	1-30	3	0-4
Information about the health system in which you work	10 (62%)	7	2-30	5	0-20
Tools for application (eg, algorithms, flow charts, checklists)	5 (31%)	4	2-10	4	0-15
Videos	7 (44%)	5	1-10	2.5	0-5

* *More than one answer was possible for this question*

♣*Not presented across WHO regions because in many cases there is one response only, where we cannot present a median*

Table S5. Engagement of Target Audience

*♣**Approaches to engage selected groups** **n = 16 (%)**	**Health-system policymakers**	**Social-system policymakers**	**Policymakers in central agencies**	**Managers of regions, central agencies**	**Stakeholders**	**Citizens/ patients**	**Other target users**
Organizational governance	13 (81%)	6 (34%)	10 (63%)	11 (69%)	9 (56%)	3 (19%)	2 (13%)
Working groups or project steering committees	14 (88%)	8 (50%)	12 (75%)	12 (75%)	12 (75%)	2 (13%)	3 (19%)
Conduct interviews to identify insights	10 (63%)	7 (44%)	7 (44%)	9 (56%)	9 (56%)	3 (19%)	3 (19%)
Reviews of draft reports	15 (94%)	7 (44%)	10 (63%)	11 (69%)	8 (50%)	2 (13%)	3 (19%)

As participants in other activities	14 (88%)	7 (44%)	8 (50%)	10 (63%)	10 (63%)	5 (31%)	5 (31%)
Other mechanism	2 (13%)	1 (6%)	2 (13%)	0	1 (6%)	1 (6%)	0

* *More than one answer was possible for this question*

♣*Not presented across WHO regions because in many cases there is one response only, where we cannot present a median*
